# The species of *Timarcha* Samouelle, 1819 described by Linnaeus (Coleoptera, Chrysomelidae)

**DOI:** 10.3897/zookeys.986.57158

**Published:** 2020-11-05

**Authors:** José Miguel Vela, Miguel Ángel Alonso-Zarazaga, Mauro Daccordi

**Affiliations:** 1 Instituto Andaluz de Investigación y Formación Agraria y Pesquera (IFAPA), Agriculture Entomology Lab, Cortijo de la Cruz, 29140, Churriana, Málaga, Spain Instituto Andaluz de Investigación y Formación Agraria y Pesquera Málaga Spain; 2 Colección de Entomología, Museo Nacional de Ciencias Naturales (CSIC), José Gutiérrez Abascal, 2, E-28006, Madrid, Spain Museo Nacional de Ciencias Naturale Madrid Spain; 3 c/o Museo Civico di Storia Naturale di Verona, Lungadige Porta Vittoria, 9, 37129, Verona, Italy c/o Museo Civico di Storia Naturale di Verona Verona Italy

**Keywords:** Chrysomelinae, Europe, leaf beetles, nomenclature, North Africa, synonyms, taxonomy, Tenebrionidae

## Abstract

Linnaeus described five species presently included in the genus *Timarcha*: *Chrysomela goettingensis*, *Tenebrio caeruleus*, *Tenebrio laevigatus*, *Tenebrio latipes*, and *Tenebrio rugosus*. After a study of the relevant material, the identity of these species has been established. The following synonyms are proposed or confirmed: *Timarcha goettingensis* (Linnaeus, 1758) = *T. latipes* (Linnaeus, 1767), **syn. nov.**; *Timarcha caerulea* (Linnaeus, 1758), **comb. nov.** = *T. balearica* Gory, 1833, **syn. nov.** = *T. balearica* Pérez Arcas, 1865, **syn. nov.**; *Timarcha rugosa* (Linnaeus, 1767) = *T. scabra* (Olivier, 1807), **syn. conf.** = *T. generosa* Erichson, 1841, **syn. conf.**; *Timarcha laevigata* (Linnaeus, 1767) = *T. tenebricosa* (Fabricius, 1775), **syn. conf.**. The type of *Tenebrio caeruleus* is a Chrysomelidae currently belonging to genus *Timarcha* and therefore can no longer be considered a Tenebrionidae (*Helops caeruleus*) nor the type species of genus *Helops*. For the sake of nomenclatural stability, an application to the International Commission on Zoological Nomenclature to change the relative precedence of *Timarcha caerulea* and retain usage of *T. balearica* will be made. An application to change the relative precedence of *Timarcha laevigata* has been submitted, which would lead to the conservation of usage of *T. tenebricosa* as valid. Lectotypes are designated for *Chrysomela goettingensis*, *Tenebrio latipes*, *Tenebrio caeruleus*, *Timarcha balearica* Gory, *T. balearica* Pérez Arcas, *Tenebrio rugosus*, *Chrysomela scabra*, *Timarcha generosa*, *Tenebrio laevigatus*, and *Chrysomela tenebricosa*. For each of the valid species the diagnosis, distribution, and host-plant data are reported.

## Introduction

The taxonomy of the genus *Timarcha* Samouelle, 1819 (Coleoptera, Chrysomelidae) is among the most challenging of all Palaearctic chrysomelids because: i) the types were rarely consulted by authors, and ii) there exists a high variability in traits as size, sculpture and form of the pronotum (Petitpierre 1970; Tiberghien 1971; Gómez-Zurita 2008; Kippenberg 2010). Therefore, a revisionary work on this genus is required (Daccordi et al. 2020).

As a starting point for the revision of the genus *Timarcha*, we have studied the species authored by Carl Linnaeus. He described five species presently belonging to this genus. They are *Chrysomela goettingensis* Linnaeus, 1758, *Tenebrio caeruleus* Linnaeus, 1758, *Tenebrio laevigatus* Linnaeus, 1767, *Tenebrio latipes* Linnaeus, 1767, and *Tenebrio rugosus* Linnaeus, 1767. One of us (MAAZ) studied and photographed the types of *T. latipes* and *T. laevigatus* in the collection of the Linnean Society of London. As well, consultation of photographs of Linnean types on the website of the Linnean Collections (http://linnean-online.org/) of the Linnean Society of London, together with a study of type specimens of other nominal species in other museums, led us to reconsider the availability and nomenclatural status of the five species of *Timarcha* described by Linnaeus.

## Material and methods

Measurements of body length were made using the ocular grid of a Lomo MBS-10 binocular microscope at 10× magnification. Body size was considered the total length of the specimen from the anterior region of head to the apex of elytron. Photographs of type specimens of *Tenebrio laevigatus* and *T. latipes* were taken with a Canon EOS 7D camera attached to a MP-E 65 mm f/2.8 1–5× macro lens. Photographs of type specimens of *Chrysomela goettingensis*, *Tenebrio caeruleus*, and *T. rugosus* were kindly provided by Linnean collections staff (The Linnean Society of London) and of *Timarcha balearica* Gory, 1833 by Antoine Mantilleri (MNHN). Types, and their parts, of *Timarcha balearica* Pérez-Arcas, 1865, *T. scabra* (Olivier, 1807), and *T. generosa* Erichson, 1841 were photographed with an Olympus Stylus TG-3 digital compact camera. Photographs of other specimens or their parts were done with a Canon EOS 550D attached to a bellows with a Schneider Componon-S 50mm f/2.8 objective. Combine ZM was used for resolving the stack of photos.

The methodology to name the vestiture under of the tarsi I–III, to dissect the sclerites of endophallus, and to inflate the endophallus is explained by Daccordi et al. (2020).

In the treatment of type material from the collection of Carl Linnaeus, we have followed Recommendation 73F (International Commission on Zoological Nomenclature 1999) and have designated “a lectotype rather than assume a holotype”. The designation of lectotypes in this paper has been made by the three authors jointly, unless otherwise indicated in the labels of the types.

Host plants are given using their valid names. If a name, now a synonym, was originally mentioned, this follows the valid name between round brackets. Plant nomenclature follows APG IV (2016) for families and The Plant List (http://www.theplantlist.org) for genera and species names.

The material examined is housed in the following collections (curators mentioned between round brackets):

**LSUK** The Linnean Collections of the Linnean Society, London (Isabelle Charmantier, Suzanne Ryder)

**MNCN**Museo Nacional de Ciencias Naturales, Madrid (Mercedes París)

**MNHN** Muséum National d’Histoire Naturelle, Paris (Antoine Mantilleri)

**ZMHB** Museum für Naturkunde der Humboldt-Universität, Berlin (Johannes Frisch, Bernd Jäger)

**ZMUK** Zoologisches Museum, Universität Kiel, Kiel (Michael Kuhlmann).

The label data for all type specimens is cited as follows: a double slash (//) divides the texts on different labels, a single slash (/) divides the text in different rows. Type localities are cited with their original spellings. Comments and notes are cited in square brackets: [p] preceding data are printed, [h] preceding data are handwritten, [w] white label, [r] red label.

The webpage of the Linnean Collections (http://linnean-online.org/), of the Linnean Society of London, has been a critical source of information.

## Results

### Timarcha goettingensis

Taxon classificationAnimaliaColeopteraChrysomelidae

(Linnaeus, 1758)

BEC2FA89-45DE-5AEC-8EFC-BCB7BDC112F2

Figures 1–4 , 5–7

Chrysomela goettingensis Linnaeus 1758: 368 (original description).
Tenebrio latipes Linnaeus 1767: 678 (original description), syn. nov.

#### Type localities.

*Chrysomela goettingensis*: “Germania”. *Tenebrio latipes*: “Africa” [type locality wrong].

#### Type material.

**Figures 1–4.**
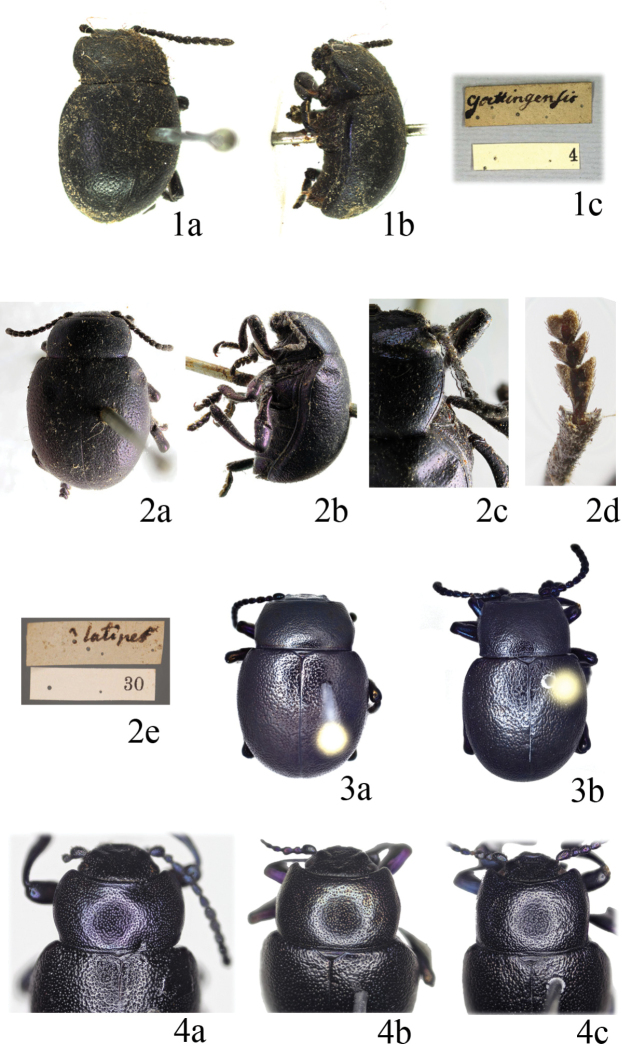
*Timarcha goettingensis***1** lectotypus of *Chrysomela goettingensis* in dorsal (**a**) and side (**b**) view, and label (**c**) (photos provided by The Linnean Society, with permission to reproduce **2** lectotypus of *Tenebrio latipes* in dorsal (**a**) and side (**b**) view, lateral side of pronotum (**c**), underside of third metatarsomere (**d**), and label (**e**) (photos by Alonso Zarazaga and Ren Li, with permission to reproduce from The Linnean Society) **3** male habitus from Germany, Frankenhausen (**a**) and Germany, Erfurt (**b**) **4** pronota of males from France, Lozère (**a**), Germany, Bad Frankenhausen (**b**) and Germany, Erfurt (**c**).

*Chrysomela goettingensis*: not examined. The images of the ***lectotype*** (♂, presently designated, Fig. 1), labelled “goettingensis [w, h, Linnaeus’ handwriting] // 4 [w, p]” (LSUK, code LINN 5537), are available at http://linnean-online.org/22922/.

*Tenebrio latipes*: ***Lectotype*** (♀, presently designated, Fig. 2): “LSL INS 6579 [p] // latipes [h, Linnaeus’ handwriting] // 30” (LSUK, code LINN 6579). Examined by one of us (MAAZ), images are also available at http://linnean-online.org/23904/.

#### Comments.

In the Linnean collections there are two different species under *Chrysomela goettingensis*. One of them, specimen LINN 5537 labelled “goettingensis” [w, h, Linnaeus’ handwriting] (http://linnean-online.org/22922/), is the lectotype of *Chrysomela goettingensis* Linnaeus, 1758: 368 (presently in *Timarcha*, Fig. 1). The other species have the codes LINN 5536 (labelled “goettingensis” [w, h, Linnaeus’ handwriting]) (http://linnean-online.org/22921/), LINN 5538 (no labelled) (http://linnean-online.org/22923/), LINN 5539 (no labelled) (http://linnean-online.org/22924/), and are to be considered syntypes of *Chrysomela goettingensis* Linnaeus, 1760: 160, although this species is currently known as *Chrysolina sturmi* (Westhoff 1882: 268) (see Waterhouse 1864: 18; Weise 1916: 96; Bieńkowski 2001: 162). An additional specimen, LINN 5540 (http://linnean-online.org/22925/), is also the latter species but cannot be a syntype because it comes from Fenwick Skrimshire, who was born after the publication of Linnaeus’s work.

Authors such as Weise (1916: 207), Winkler (1930: 1298), Bechynĕ (1945a: 103, 1947a: 59, 1948: 50), Jolivet (1967a: 225); Warchałowski (2010: 629), and Gómez-Zurita and Kippenberg (2010: 440 (pars)) have identified as “*Timarcha latipes* (Linnaeus)” specimens belonging to *T. punctella* Marseul, 1871 species group (Daccordi and Vela unpubl. data). However, the original description of *Tenebrio latipes* Linnaeus, 1767 clearly says that it is half the size of *T. laevigata*. In fact, the lectotype of *T. latipes* measures 8.4 mm (Fig. 2), obviously much smaller than *T. punctella* or species similar to it from North Africa. The type locality “Africa” given by Linnaeus (1867) for *T. latipes* is incorrect.

#### Diagnosis.

**Figures 5–7.**
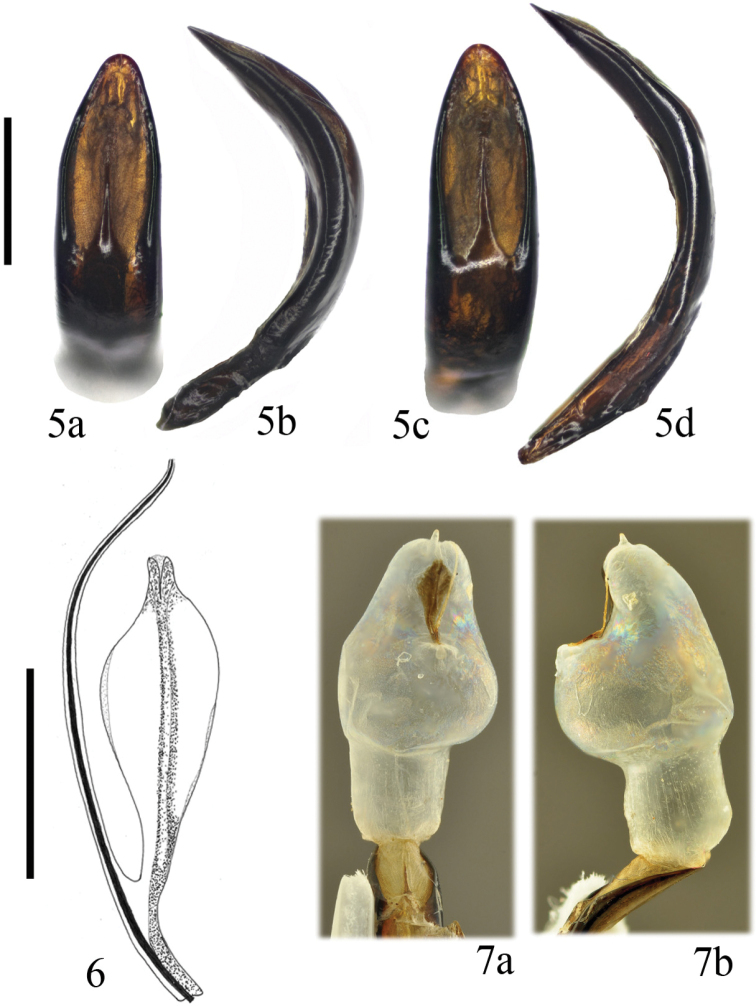
*Timarcha goettingensis***5** aedeagi of Germany, Erfurt in dorsal (**a**) and side view (**b**) and Germany, Bad Frankenhausen in dorsal (**c**) and side (**d**) view **6** sclerite of the endophallus from Germany, Bad Frankenhausen, in dorsolateral view (taken from Daccordi et al. 2020) **7** everted endophallus from Germany, Frankenhausen in dorsal (**a**) and side (**b**) view (taken from Daccordi et al. 2020). Scale bars: 1 mm (**5**), 0.5 mm (**6**).

Males: 7.4–12.6 mm (lectotype of *T. goettingensis*: 8.7 mm; Fig. 1); females: 8.4–14.5 mm (lectotype of *T. latipes* is an unextended specimen measuring 8.4 mm; Fig. 2). Black or black with bluish luster (Fig. 3). Highly variable species in brightness, puncturation, form of the pronotum and elytra, and size. Sides of the pronotum regularly, slightly curved or almost straight, with the widest point in the basal third or at base, never cordiform, completely margined or with lateral margins obliterated at different extent (Fig. 4). Puncturation on the pronotum and elytra dense, regular, heavily or weakly marked, usually stronger on the elytra, not or conspicuously vermiculated (Figs 3, 4). Mesoventrite variable with apophysis a bit prominent, slightly forked, or more or less emarginated or almost straight, never clearly bituberculated. Vestiture of the female tarsi: (1, 1, 1/3–3/4; 1, 1, 1/3–3/4; 1, 4/5–1, 1/2–3/4). The aedeagus is slender and progressively narrowed towards the apex in dorsal view and regularly curved in side view (Fig. 5). Sclerites of the internal sac of the aedeagus (Fig. 6) with a paired phanera in romboid form, which is an important diagnostic character to separate this from other closely related species. The inflated endophallus of an approximate locotype (coming near Göttinga in central Germany) (Fig. 7) is shown. A number of species and subspecies, whose taxonomical rank has yet to be studied, have been described in association with this species (Winkelman and Debreuil 2008; Warchałowski 2010; Gómez-Zurita and Kippenberg 2010).

#### Distribution.

Most of Europe, from northern Spain to European Russia, and reaching Great Britain and Sweden (Gómez-Zurita and Kippenberg 2010)

#### Host plants.

*Scabiosa atropurpurea* L. (= *S. maritima* L.) (Caprifoliaceae); *Plantago lanceolata* L., *P. coronopus* L. (Plantaginaceae), *Cruciata laevipes* Opiz, *Galium aparine* L., *G. arenarium* Loisel., *G. mollugo* L., *G. odoratum* (L.) Scop. (= *Asperula odorata* L.), *G. saxatile* L., *G. uliginosum* L., *G. verum* L., *Rubia peregrina* L. (Rubiaceae) (Jolivet and Petitpierre 1973; Winkelman and Debreuil 2008; Tiberghien 2016).

### Timarcha balearica

Taxon classificationAnimaliaColeopteraChrysomelidae

Gory, 1833

42AE1909-04C7-5EE1-9E36-67415479466E

Figures 8–14

Tenebrio caeruleus Linnaeus 1758: 418 (original description), syn. nov. Application for reversal of precedence will be submitted to the International Commission on Zoological Nomenclature (see comments below).
Tenebrio caeruleus Linnaeus 1767: 677 (repeated description).
Timarcha caerulea (Linnaeus 1758), nov. comb.
Timarcha balearica Gory 1833: pl. 49 (original description).
Timarcha balearica Gory 1844: 300 (text description).
Timarcha balearica Pérez Arcas 1865: 180 (original description). Synonymized with T. balearica Gory by Fairmaire and Allard (1873: 152). Synonymy confirmed.
Timarcha balearica var. violaceus Pic 1919: 20 (unavailable infrasubspecific name).
Timarcha balearica var. martini Pic 1919: 20 (unavailable infrasubspecific name).
Timarcha balearica ab. viridipennis Bechynĕ 1946: 30 (unavailable infrasubspecific name).
Timarcha balearica ab. coerulescens Bechynĕ 1946: 30 (unavailable infrasubspecific name).
Timarcha balearica ab. longicornis Bechynĕ 1946: 30 (unavailable infrasubspecific name).
Timarcha balearica ab. nigriventris Bechynĕ 1946: 30 (unavailable infrasubspecific name).
Timarcha balearica ab. olivacea Bechynĕ 1946: 30 (unavailable infrasubspecific name).
Timarcha balearica ab. semicoerulea Bechynĕ 1946: 30 (unavailable infrasubspecific name).
Timarcha balearica ab. discolor Bechynĕ 1946: 30 (unavailable infrasubspecific name).
Timarcha balearica ab. tricolor Bechynĕ 1946: 30 (unavailable infrasubspecific name).

#### Type localities.

*Tenebrio caeruleus*: “Hispania”. *Timarcha balearica* G.: “Les Iles Baléares”. *Timarcha balearica* P. A.: “Mahón (Menorca), Alcudia de Mallorca”.

#### Type material.

**Figures 8–14.**
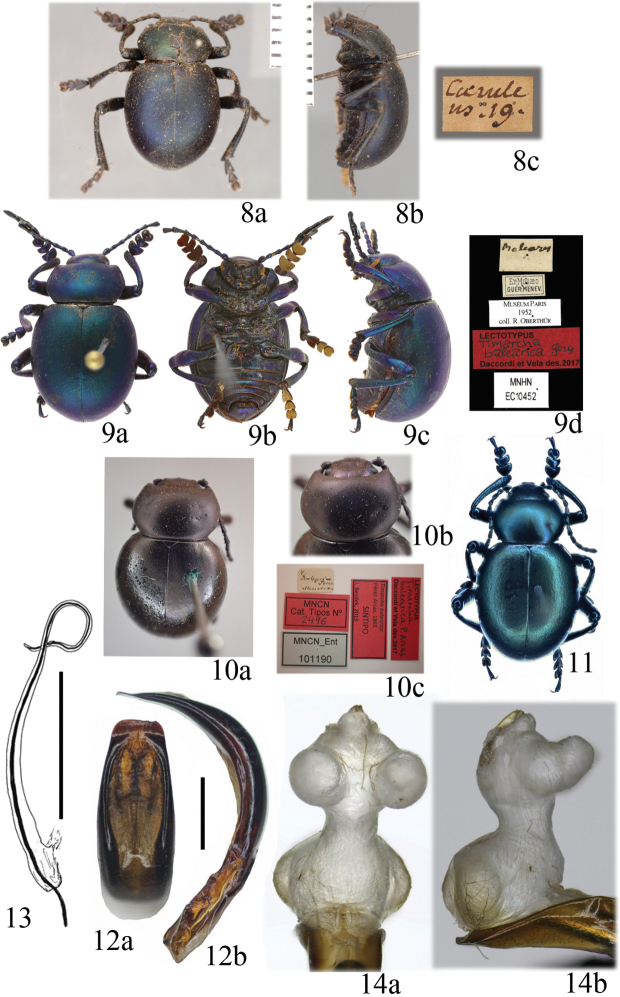
*Timarcha balearica***8** lectotypus of *Tenebrio caeruleus* in dorsal (**a**) and side (**b**) view, and label (**c**) (photos provided by The Linnean Society, with permission to reproduce) **9** lectotypus of *Timarcha balearica* Gory in dorsal (**a**), ventral (**b**) and side (**c**) view, and label (**d**) (photos provided by Antoine Mantilleri (MNHN), with permission to reproduce) **10** lectotypus of *Timarcha balearica* Pérez Arcas, habitus (**a**), pronotum (**b**) and labels (**c**) **11** male habitus from Spain, Baleares, Palma de Mallorca **12** aedeagus from Spain, Baleares, Palma de Mallorca, in dorsal (**a**) and side (**b**) view **13** sclerite of the endophallus from Spain, Palma de Mallorca, in side view (taken from Daccordi et al. 2020) **14** Everted endophallus from Spain, Baleares, Menorca, Mahón, in dorsal (**a**) and side (**b**) view. Scale bars: 1 mm (**12**), 0.5 mm (**13**).

*Tenebrio caeruleus*: not examined. The images of the ***lectotype*** (♂, presently designated, Fig. 8), labelled “coerule / us 19’ [w, h, Linnaeus’s handwriting]” (LSUK, code LINN 6569), are available at http://linnean-online.org/23894/.

*Timarcha balearica* G.: ***Lectotype*** (♂, presently designated, Fig. 9): “Baleares [h. by Blanchard] // Ex-Musaeo / GUÉR.-MÉNEV. [p, w] // ***lectotypus*** [p] / Timarcha / balearica Gory [h] / Daccordi et Vela des. 2017 [p, r]”. ***Paralectotypes***: 2 ♂♂: same label text as lectotype, but ***paralectotypus*** instead of ***lectotypus*** (MNHN, Col. Oberthür).

*Timarcha balearica* P.-A.: ***Lectotype*** (♂, presently designated, Fig. 10): “T. / Balearica / Perez / Menorca [h. by Pérez Arcas,w] // MNCN / Cat. Tipos N° / 2496 [p, r] // MNCN_Ent / 101190 [p, grey] // Timarcha balearica / Pérez Arcas, 1865 / SINTIPO / J. Bezdek, 2013 [p, r] // ***lectotypus*** [p] / Timarcha / balearica P. Arcas [h] / Daccordi et Vela des. 2017 [p, r]” (MNCN).

#### Comments.

*Timarcha balearica* was described for the first time as figure 8 in planche 49 (Gory 1833). Later, Gory (1844: 300) published a text description (see Bousquet 2016 for exact publication dates).

Linnaeus (1758: 418) described *Tenebrio caeruleus* (Fig. 8; here considered a synonym of *Timarcha balearica*), with these words: “T. apterus caerulescens, thorace suborbiculato, coleoptris obtusis. Habitat in Hispania”. Some years later, Linnaeus (1764: 98) made an extended description expanding the locality to “Europa australiore” and provided more characters: “Corpus magnitudine, colore, statura & facie T. mortisagi, sed. Antennae caeruleae, apice nigrae, nec totae nigrae. Thorax brevior, postice parum rotundatus, nec postice truncates. Elytra marginibus lateralibus atro-caerulescentibus, apice obtuso nec acuminato. Femora atro-caerulescentia, nitida, nec nigra opaca”. Later, Linnaeus (1767: 677) turned back repeating exactly the description of 1758, but not that of 1764.

Fabricius (1775: 257) proposed the combination *Helops caeruleus* (Coleoptera, Tenebrionidae) for a beetle from “Europa australi”, making a reference to the Linnaean descriptions of 1758 and 1764, but adding “elytris striatis” and “antennae pedesque nigrae” to the description of Linnaeus (1758: 418); these characters are clearly not found in the type of *Tenebrio caeruleus* Linnaeus, 1758, where the elytra are smooth and legs are bluish. To date, the type species of the genus *Helops* (Coleoptera, Tenebrionidae) is *Tenebrio caeruleus* Linnaeus, 1758 (Nabozhenko et al. 2008; International Commission on Zoological Nomenclature 2009), but this statement should be changed as most probably *Helops caeruleus* was described by Fabricius, not by Linnaeus (in *Tenebrio*). Interestingly, Illiger (1802: 410) rightly stated that *Tenebrio caeruleus* should be considered as belonging to genus *Chrysomela* (genus *Timarcha* was not described until 1819 by Samouelle). However, since 1802 no one has mentioned *Tenebrio caeruleus* as a Chrysomelidae.

For the sake of stability (Art. 23.2, International Commission on Zoological Nomenclature 1999), it would be convenient to apply the reversal of precedence and declare *Timarcha balearica* Gory, 1833 a *nomen protectum*. The requirements of Art. 23.9.1.2 are met by quoting the following references: Jolivet (1967b, 1995), Petitpierre (1970, 1973, 1985, 2011), Jolivet and Petitpierre (1973, 1981), Petitpierre et al. (1993), Chevin (1994), Petitpierre and Juan (1994), Santiago-Blay and Fain (1994), Steinhausen (1994), Jolivet and Hawkeswood (1995), Jolivet (1998), Teunissen (2002), Warchałowski (2003, 2010), Gómez-Zurita (2004, 2008), Gómez-Zurita et al. (2000, 2004), Gómez-Zurita and Galián (2005), Davison and Blaxter (2005), Jolivet and Poinar (2007), Gómez-Zurita and Kippenberg (2010), Mravinac et al. (2011), Jolivet et al. (2014), Petitpierre and Anichtchenko (2018), Petitpierre (2019), Daccordi et al. (2020). However, the name *Tenebrio caeruleus* Linnaeus, 1758 does not meet the requirements of Art. 23.9.1.1, because, until the present, it has been used in its misinterpreted concept of a Tenebrionidae of genus *Helops*. Therefore, an application is to be submitted to the International Commission of Zoological Nomenclature in order to maintain usage of *T. balearica* as a valid species, under Art. 23.9.3. Nomenclatural stability would be negatively affected by using *Tenebrio caerulea* Linnaeus (presently combined in *Timarcha*) as a valid name owing to its current ambiguity.

#### Diagnosis.

Males: 12.0–14.5 mm (lectotype of *Tenebrio caeruleus*: 12.2 mm, Fig. 8; lectotype of *T. balearica* Gory: 12.7 mm, Fig. 9; lectotype of *T. balearica* P. Arcas: 12.0 mm, Fig. 10); females: 14.9–17.3 mm. Coloration variable from black, greenish, bluish, or copper-violet, or a combination; 11 color variations have been described (Pic 1919; Bechynĕ 1946; Compte 1956). Lateral sides of the pronotum curved, narrower at base; margin conspicuous on all four sides except in lateral sides near the base, where it is obliterated. Pronotum and elytra smooth, puncturation absent (Fig. 11). Mesoventrite divergently bituberculated. Vestiture tarsal formulae: ♂♂ (0, 0, 0; 0, 0, 0; 1/3, 0, 0), ♀♀ (1/4, 0, 0; 1/4–1/3, 0, 0; 3/4, 0, 0), very distinctive in females. Aedeagus very characteristic in its truncate apex in dorsal view; in side view it is curved in its second half (Fig. 12). Sclerite of internal sac of aedeagus with a much reduced phanera and a looped flagellum (Fig. 13; see also Petitpierre 1970: fig. 8 and Petitpierre 2019: fig. 19). The inflated endophallus is as illustrated (Fig. 14; see also Petitpierre and Anichtchenko 2018: fig. 11).

#### Distribution.

Balearic Islands: Mallorca and Menorca (Tenenbaum 1915; Jolivet 1953; Compte 1956).

#### Host plants.

*Rubia peregrina* L. (= *R. angustifolia* L.), *Galium* spp., *Asperula* spp. (Rubiaceae) (Jolivet 1953; Jolivet and Petitpierre 1973; Jolivet and Poinar 2004), *Plantago lanceolata* L. (Plantaginaceae) (Petitpierre 1985).

### Timarcha rugosa

Taxon classificationAnimaliaColeopteraChrysomelidae

(Linnaeus, 1767)

0FEF0D49-2B1F-567B-8BB4-E08CAD14F238

Figures 15–17 , 18–22

Tenebrio rugosus Linnaeus 1767: 678 (original description).
Chrysomela scabra Olivier 1807: 507 (original description). Synonymized by Fairmaire (1884: 89). Synonymy confirmed.
Timarcha generosa Erichson 1841: 189 (original description). Synonymized by Fairmaire and Allard (1873: 161). Synonymy confirmed.

#### Type localities.

*Tenebrio rugosus*: “Africa” [other localities mentioned in the original description as “Hispania” and “Gallia” are erroneous and should not be taken into consideration following Recommendation 76A.2 of the Code (International Commission on Zoological Nomenclature 1999)]. *Chrysomela scabra*: “côte de Barbarie”. *Timarcha generosa*: “Bona”.

#### Type material.

**Figures 15–17.**
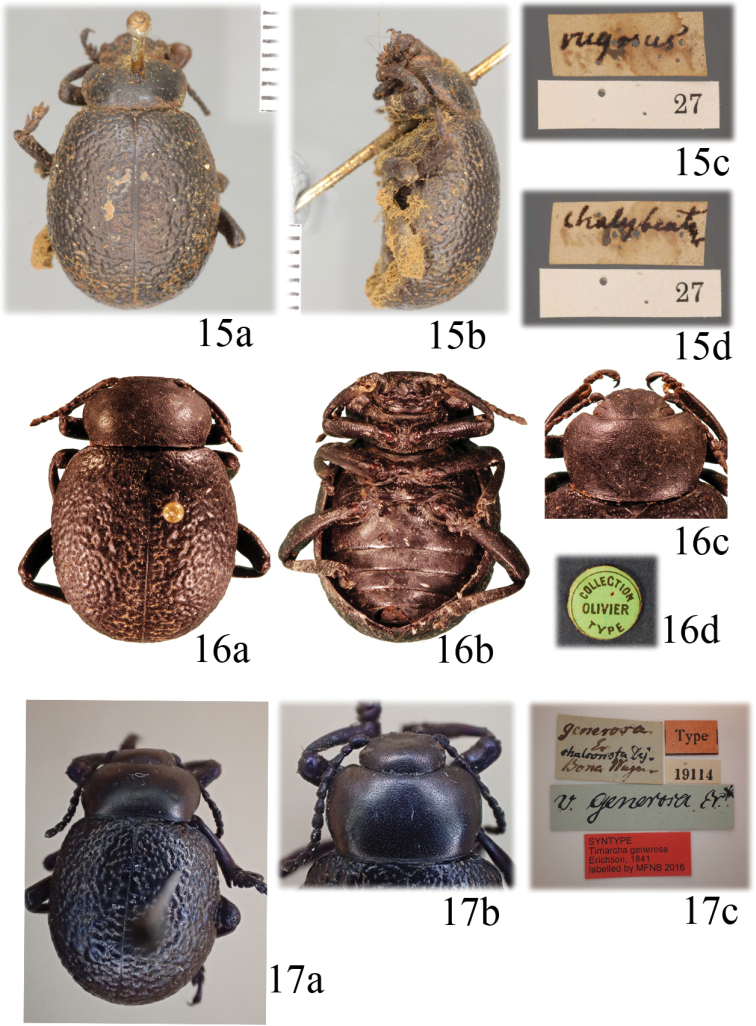
*Timarcha rugosa***15** lectotypus of *Tenebrio rugosus* in dorsal (**a**) and side (**b**) view, and label in upper (**c**) and lower view (**d**) (photos provided by The Linnean Society, with permission to reproduce) **16** lectotypus of *Timarcha scabra* in dorsal (**a**), and ventral (**b**) view, pronotum (**c**) and label (**d**) **17** Lectotypus of *Timarcha generosa* in dorsal view (**a**), pronotum (**b**) and label (**c**).

*Tenebrio rugosus*: not examined. The photographs of the ***lectotype*** (♀, presently designated, Fig. 15), labelled “rugosus / chalybeata [reversal, w, h, Linnaeus’ handwriting] // 27 [w, p]” (LSUK, code LINN 6576), are available at http://linnean-online.org/23901/.

*Chrysomela scabra*: ***Lectotype*** (♀, presently designated, Fig. 16): “COLLECTION / OLIVIER / TYPE [round green label, p] // ***lectotypus*** [p] / Timarcha / scabra Olivier [h] / Daccordi et Vela des. 2017 [p, r] // Timarcha [p] / rugosa L. [h] / Daccordi et Vela det. 2017 [p, w]” (MNHN).

*Timarcha generosa*: ***Lectotype*** (♂, presently designated, Fig. 17): “generosa / Er. / chalconota Dej. / Bona Wagner [h, w] // v. generosa Er. [h, bluish label] // 19114 [p, w] // Type [p, r] // ***syntype*** / Timarcha generosa / Erichson, 1841 / labelled by MFNB 2016 [p, r] // ***lectotypus*** [p] / Timarcha / generosa Erichson [h] / Daccordi et Vela des. 2017 [p, r]” (ZMHB). ***Paralectotype***: 1 ♂ “Hist.-Coll. (Coleoptera) / Nr. 19114 / Timarcha generosa Erichs. / Bona, Wagner / Zool. Mus. Berlin [p, w] // 19114 [p, w] // Type [p, r] // ***syntype*** / Timarcha generosa / Erichson, 1841 / labelled by MFNB 2016 [p, r] // ***paralectotypus*** [p] / Timarcha / generosa Erichs. [h] / Daccordi et Vela des. 2017 [p, r]” (ZMHB). ***Paralectotype***: 1 ♀ “Hist.-Coll. (Coleoptera) / Nr. 19114 / Timarcha generosa Erichs. / Bona, Wagner / Zool. Mus. Berlin [p, w] // 19114 [p, w] // Type [p, r] // ***syntype*** / Timarcha generosa / Erichson, 1841 / labelled by MFNB 2016 [p, r] // ***paralectotypus*** [p] / Timarcha / generosa Erichs. [h] / Daccordi et Vela des. 2017 [p, r]” (ZMHB). All the specimens carry a label: “TIMARCHA [p] / rugosa L. [h] / Daccordi et Vela det. 2017 [p, w].

#### Comments.

Fairmaire (1884: 89) and Fairmaire and Allard (1873: 161), respectively, considered *Chrysomela scabra* and *Timarcha generosa* as junior synonyms of *T. rugosa*, and we can confirm these decisions. However, since Bechynĕ (1947a: 56) to present, *T. generosa* and *T. scabra* were regarded as separate species (Gómez-Zurita and Kippenberg 2010: 439, 441; Warchałowski 2010: 625). The lectotype of *T. generosa* designated herein has blackish legs, but it is interesting that the two paralectotypes have reddish legs, showing this color variation which is not uncommon in several *Timarcha* species.

#### Diagnosis.

**Figures 18–22.**
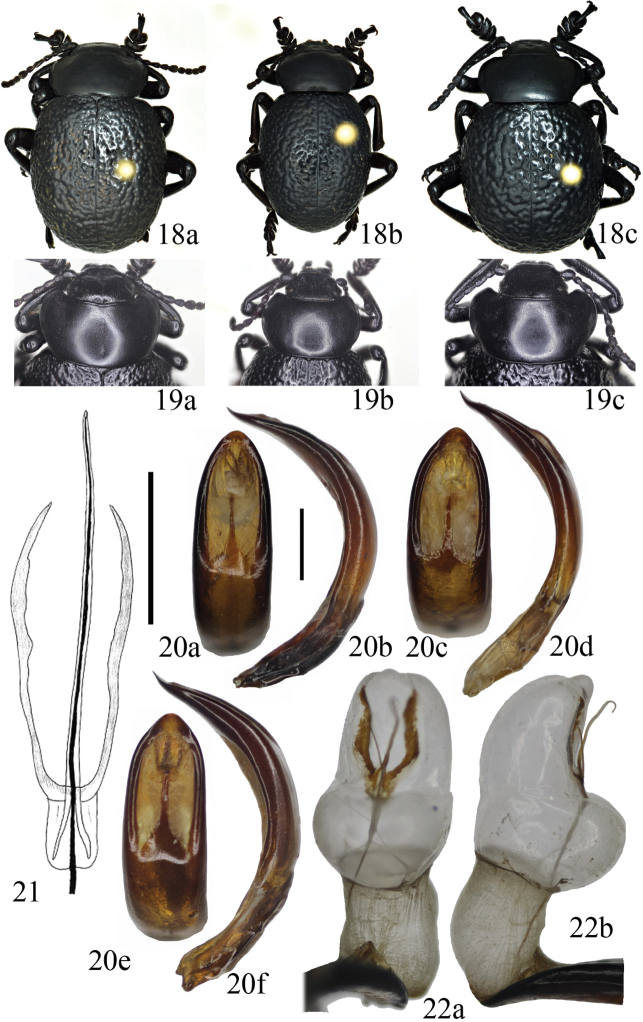
*Timarcha rugosa***18** male habitus from Algery, Mandoura (**a**), Morocco, Debdou (**b**) and Morocco, Aguelmame Sidi Ali (**c**) **19** pronota of males from Algery, Mandoura (**a**), Morocco, Debdou (**b**) and Morocco, Aguelmam Sidi Ali (**c**) **20** aedeagi from Morocco, Oujda in dorsal (**a**) and side (**b**) view, Morocco, Aguelmame Sidi Ali in dorsal (**c**) and side (**d**) view and Morocco, Annual in dorsal (**e**) and side (**f**) view **21** sclerite of the endophallus from Algery, Batna in dorsal view (taken from Daccordi et al. 2020) **22** Everted endophallus from Morocco, Ain Benimathar, in dorsal (**a**) and side view (**b**). Scale bars: 1 mm (**20**), 0.5 mm (**21**).

Males: 11.8–19.0 mm (lectotype of *T. generosa*: 15.8 mm, Fig. 17); females: 13.7–21.2 mm (lectotype of *Tenebrio rugosus* is an extended specimen measuring 23.5 mm, Fig. 15; lectotype of *Timarcha scabra* is an unextended specimen measuring 15.6 mm, Fig. 16). Species variable in size, form of pronotum, and elytral sculpture. Black, shining or matte, sometimes with bronze tan. Legs black or femora and tibiae reddish, also antennomeres I–V can be reddish at base in populations of northern Algeria. Pronotum cordiform or subcordiform, with maximum width at distal 1/3, reborded even at posterior angles, without or with weak punctures which are not very dense (Fig. 19). Elytra not or weakly punctured, always conspicuously vermiculate, giving a rugose aspect (Fig. 18). Mesoventrite straight or weakly emarginate, not or weakly prominent. In ventral view, meso- and metatarsomere III slightly emarginated at apex in males; in females, this emargination is well marked, which is a differential feature relative to other species. Vestiture tarsal formulae: ♂♂ (0,0,0; 0–1/3,0,0; 0–4/5 (very finely),0,0), ♀♀ (1, 1, 1; 1, 1, 1; 1, 1, 1). Aedeagus somewhat variable, generally broad (Fig. 20c, e) but sometimes narrower (Fig. 20a) in dorsal view, and also more or less curved in side view (Fig. 20b, d, f). Sclerites of internal sac of aedeagus, in dorsoventral view, with wide or fine, slightly curved, and paired phanera, and a straight flagellum (Figs 21, 22). The inflated endophallus is shown in Figure 22.

#### Distribution.

Tunisia, Algeria, and Morocco. Spain and France, as in the original description of *T. rugosus*, are wrong.

#### Host plants.

*Plantago albicans* L. (Plantaginaceae), *Asperula* sp. (Rubiaceae) (Jolivet 1966).

### Timarcha tenebricosa

Taxon classificationAnimaliaColeopteraChrysomelidae

(Fabricius, 1775)

F8AD6D54-2F7B-5600-87AD-098E94D7520D

**Figures 23, 24.**
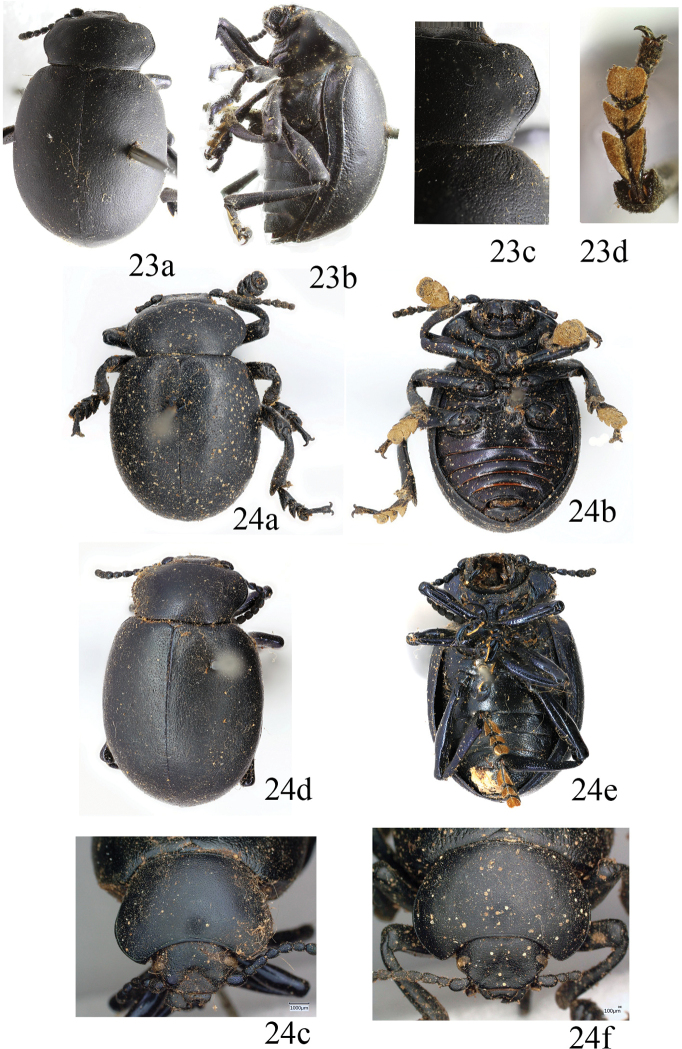
*Timarcha tenebricosa***23** lectotypus of *Tenebrio laevigatus* in dorsal (**a**) and side view (**b**), pronotum (**c**) and metatarsus in lower view (**d**) (photos by Alonso Zarazaga and Ren Li, with permission to reproduce from The Linnean Society) **24** typi of *Chrysomela tenebricosa* Fabricius, lectotypus male in dorsal (**a**) and ventral (**b**) view, and pronotum (**c**), paralectotypus female in dorsal (**d**) and ventral (**e**) view, and pronotum (**f**) (photographed by Michael Kuhlmann, Zoologischen Museum Kiel, reproduced with permission).

**Figures 25–29.**
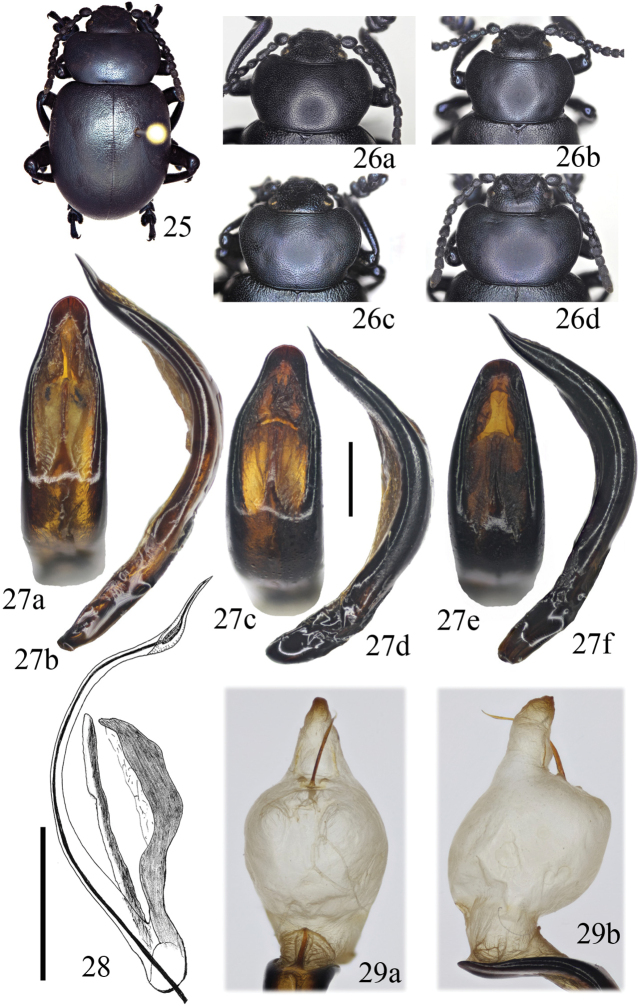
*Timarcha tenebricosa***25** male habitus from France, Poitiers **26** pronota of males of males of Crimea (**a**), Austria, Vienna (**b**), France, Paimport (**c**) and France, Poitiers (**d**) **27** aedeagi from France, Alps Maritimes, Caussols in dorsal (**a**) and side (**b**) view, France, Normandie, Bréal in dorsal (**c**) and side (**d**) view, Crimea in dorsal (**e**) and side (**f**) view **28** sclerite of the endophallus from England, Launceston in dorsolateral view (taken from Daccordi et al. 2020) **29** Everted endophallus from France, Paimport in dorsal (**a**) and side view (**b**). Scale bars: 1 mm (**27**), 0.5 mm (**28**).

Figures 23–24 , 25–29

Tenebrio laevigatus Linnaeus 1767: 678 (original description), syn nov. Application for reversal of precedence submitted to the International Commission on Zoological Nomenclature (see comments below).
Tenebrio coeruleus Berkenhout 1769: 111 (non T. caeruleusLinnaeus 1758: 418). Synonymized with T. tenebricosa by Stephens (1829: 224).
Chrysomela tenebricosa Fabricius 1775: 94 (unjustified replacement name). Synonymized explicitly with T. laevigatus by Duftschmid (1825: 161) and Stephens (1831: 348).
Chrysomela tenebriosa : Fabricius 1781: 116 (incorrect spelling).
Chrysomela tenebrioides : Gmelin 1790: 1667 (incorrect spelling).
Chrysomela tenebricosa : Olivier 1791: 689; Rossi 1790: 74; Herbst 1794: 104; Fabricius 1801: 423; Panzer 1797: 44, 1; Illiger 1802: 410; Latreille 1804: 376; Olivier 1807: 508. Schönherr 1808: 239.
Tenebrio coeruleus : Berkenhout 1795: 109.
Timarcha tenebricosa : Samouelle 1819: 213 (combination); Kirby 1826: 99; Stephens 1829: 224; Herrich-Schäffer 1838: 156, 21b; Gemminger and Harold 1871: 3462; Fairmaire and Allard 1873: 169; Weise 1882: 321, 1916: 211; Marseul 1883: 49; Heyden et al. 1883: 197; Fairmaire 1884: 93; Reitter 1913: 108; Bechynĕ 1945b: 7; Bechynĕ 1947b: 8; Müller 1952: 450; Jeanne 1967: 8; Mohr 1966: 191; Petitpierre 1970: 5, 1973: 10; Tiberghien 1971: 190, 2014: 2; Minelli and Vittorelli 1976: 20; Kippenberg 1994: 86; Lopatin et al. 2004: 83; Winkelman and Debreuil 2008: 42; Warchałowski 2003: 223, 2010: 628; Gómez-Zurita and Kippenberg 2010: 442; Petitpierre and Anichtchenko 2018: 364; Petitpierre 2019: 109.
Chrysomela laevigata : Duftschmid 1825: 161 (combination).
Timarcha laevigata : Latreille 1829: 150 (virtual combination); Stephens 1831: 348; 1839: 308; Dufour 1843: 106; Küster 1847: 91; Little 1838: 237; Shuckard and Spry 1861: 70; Steiner 1864: 208; Waterhouse 1864: 26; Brunetti 1880: 235; Cuní-Martorell and Martorell-Peña 1876: 321; Cuní-Martorell 1885: 62, 1888: 159; Apfelbeck 1907: 506.

#### Type localities.

*Tenebrio laevigatus*: “Africa” [wrong type locality]. *Chrysomela tenebricosa*: “Europa australiori”.

#### Type material.

*Tenebrio laevigatus*: ***Lectotype*** (♀, designated herein, Fig. 23): “laevigatus [h, probably by Linnaeus] // 29 [p]” (LSUK, code LINN 6578). Examined by one of us (MAAZ), images are also available at http://linnean-online.org/23903/.

*Chrysomela tenebricosa*: not examined. Syntypes (1 ♂, 1♀, Fig. 24) (ZMUK) were examined from photographs. Minelli and Vittorelli (1976) designated *in litteris* (1974) the male as the “lectoholotypus”, the female as the “lectoallotypus”. Here we formally designate the male as ***lectotype*** (Fig. 24a–c), and the female as ***paralectotype*** (Fig. 24d–f).

#### Comments.

The lectotype of *T. laevigata* (L.), a female (Fig. 23), has the pronotum and elytra finely and regularly punctured, the mesoventrite protruding and slightly bituberculated, and the metatarsus setose with the vestiture formula (1, 1/2, 1/3) (Fig. 23d); this perfectly fits specimens of the well-known European *T. tenebricosa*. On the other hand, females of *Timarcha laevigata auct. nec* Linnaeus, 1767 from North Africa, i.e. *T. turbida* Erichson, 1841: 189, or even *T. punctella* Marseul, 1871: 387, are different in that they have a pronotum with finer punctures, the mesoventrite very scarcely protruding and slightly emarginated but not bituberculate, and the female metatarsus with a large glabrous strip underside and a vestiture formula (1, 1, ½–¾) (*T. turbida*) or (1,1,1) (*T. punctella*).

As the name *Timarcha laevigata*, in the sense here fixed as a synonym of *T. tenebricosa*, has at least one usage since 1899 (in Apfelbeck 1907: 500, 502, 506), one of the two conditions required by International Code of Zoological Nomenclature (1999, Art. 23.9.1.1) for reversal of precedence is not accomplished. Besides, the name *T. laevigata*, although wrongly applied to a North African species, has been profusely used until now (see e.g. Gómez-Zurita and Kippenberg 2010; Warchałowski 2010). An application has been submitted to the International Commission of Zoological Nomenclature (Vela et al. 2018) to maintain usage of *T. tenebricosa* as a valid species. The type species of *Timarcha* Samouelle is *Chrysomela tenebricosa*Fabricius 1775, by subsequent designation by Chevrolat (1843: 655 in Löbl and Smetana 2011: 50).

#### Diagnosis.

Males: 14.6–17.2 mm; females: 16.1–18.2 mm (lectotype of *Tenebrio laevigata* = 17.5 mm; Fig. 23). Black or with bluish luster. Surface microreticulate, with a dull aspect (Fig. 25). Pronotum subcordiform, or cordiform, usually widest at the anterior 1/3, completely rebordered by a fine furrow, sides regularly curved. However, there is a much variation in the form of pronotum (Fig. 26), and the lateral sides near the base may be straight (Fig. 26a, d), sinuate (Fig. 26b), or both straight and sinuate (e.g. left side straight, right side sinuate; Fig. 26c). Puncturation on pronotum (Fig. 26) and elytra dense, regular, moderately marked, on a smooth surface never vermiculate. Mesoventrite with apophysis somewhat protruding, more or less emarginate, sometimes slightly bituberculate. Vestiture tarsal formulae: ♂♂ (0,0,0; 0,0,0; 1/3–1/2,0,0), ♀♀ (1/2,0,0; 1/2–3/4,0–1/2,0–1/2; 3/4–1,1/3–1/2,1/3–1/2). Aedeagus variable but always with paddle-shaped at the apex in dorsal view and strongly curved with sinuate apex in lateral view (Fig. 27). Sclerites of the internal sac of aedeagus with a long, curved flagellum that is somewhat widened before the apex in dorso-lateral view (Fig. 28); the phanera consist in two paired wings elongated and curved. The inflated endophallus is shown in Figure 29 (see also Petitpierre and Anichtchenko 2018: fig. 3). Thirteen subspecies have been described (Bechynĕ 1945b, 1948; Müller 1952), whose taxonomic status is very doubtful (Minelli and Vitorelli 1976; Warchałowski 2010).

#### Distribution.

Most of Europe, from northern Spain to Great Britain and Ireland, eastwards to Georgia, Azerbaijan, and Asiatic Turkey; not recorded in Scandinavia (Gómez-Zurita and Kippenberg 2010).

#### Host plants.

On Rubiaceae: *Asperula cynanchica* L., *Cruciata laevipes* Opiz, *Galium aparine* L., *G. mollugo* L., *G. parisiense* (L.), *G. verum* L., *Rubia peregrina* L. (Jolivet and Petitpierre 1973; Winkelman and Debreuil 2008).

## Discussion

The difficult task of studying the types of old species has been greatly facilitated with the quick access to high-quality images. As a result, it is now easier for taxonomists to verify the status of old synonyms or interpretations which were based upon very short, generalized descriptions. The high-quality images of the types in the Linnaean collections have proven extremely useful for the zoological community. In addition, most museums and their curators are willing to help with search for and loan of types, which can facilitate taxonomic work. Incorrect species concepts, not based on the examination of name-bearing types, have sometimes been maintained despite the identity of extant type material, which has been carefully cared for and maintained for many years for the benefit of science.

In the case of genus *Timarcha*, whose revision is very necessary, the slow and sometimes difficult work of consulting types has become absolutely necessary, as various authors have made different interpretations for a long time. Although historical misinterpretations of *T. laevigata* and *T. latipes* have ascribed these to different North African species or even to both sexes of the same species, these two species are actually two very different European species. Also, *T. caerulea* is revealing, as it was considered a Tenebrionidae, when in actuality the type is clearly a male of the genus *Timarcha*, identical to *T. balearica* (Chrysomelidae).

The main synonyms presented here can be summarized as follows:

***Timarcha goettingensis* (Linnaeus, 1758)**

= *T. latipes* (Linnaeus, 1767), syn. nov.

***Timarcha balearica* Gory, 1833 (to be proposed to ICZN as a *nomen protectum*)**

= *Timarcha caerulea* (Linnaeus, 1758), syn. nov., comb. nov. (to be proposed to ICZN as a *nomen oblitum*)

***Timarcha rugosa* (Linnaeus, 1767)**

= *T. scabra* (Olivier, 1807), syn. conf.

= *T. generosa* Erichson, 1841, syn. conf.

***Timarcha tenebricosa* (Fabricius, 1775) (proposed to ICZN as a *nomen protectum*)**

= *T. laevigata* (Linnaeus, 1767), syn. conf. (proposed to ICZN as a *nomen oblitum*)

## Supplementary Material

XML Treatment for Timarcha goettingensis

XML Treatment for Timarcha balearica

XML Treatment for Timarcha rugosa

XML Treatment for Timarcha tenebricosa
